# A Novel Nine-Gene Signature Associated With Immune Infiltration for Predicting Prognosis in Hepatocellular Carcinoma

**DOI:** 10.3389/fgene.2021.730732

**Published:** 2021-11-30

**Authors:** Rongqiang Liu, ZeKun Jiang, Weihao Kong, Shiyang Zheng, Tianxing Dai, Guoying Wang

**Affiliations:** ^1^ Department of Hepatobiliary Surgery, The First Affiliated Hospital of Guangzhou Medical University, Guangzhou, China; ^2^ Department of Gastrointestinal Surgery, The First Affiliated Hospital of Guangzhou Medical University, Guangzhou, China; ^3^ Department of Emergency Surgery, The First Affiliated Hospital of Anhui Medical University, Hefei, China; ^4^ Department of Breast Surgery, The Third Affiliated Hospital of Guangzhou Medical University, Guangzhou, China; ^5^ Department of Hepatic Surgery and Liver Transplantation Center, The Third Affiliated Hospital of Sun Yat-sen University, Guangzhou, China

**Keywords:** AURKA, gene signature, hepatocellular carcinoma, prognosis, immune infiltration, nomogram

## Abstract

**Background:** Hepatocellular carcinoma (HCC) is one of the most common malignant tumors worldwide, and its prognosis remains unsatisfactory. The identification of new and effective markers is helpful for better predicting the prognosis of patients with HCC and for conducting individualized management. The oncogene Aurora kinase A (AURKA) is involved in a variety of tumors; however, its role in liver cancer is poorly understood. The aim of this study was to establish AURKA-related gene signatures for predicting the prognosis of patients with HCC.

**Methods:** We first analyzed the expression of AURKA in liver cancer and its prognostic significance in different data sets. Subsequently, we selected genes with prognostic value related to AURKA and constructed a gene signature based on them. The predictive ability of the gene signature was tested using the HCC cohort development and verification data sets. A nomogram was constructed by integrating the risk score and clinicopathological characteristics. Finally, the influence of the gene signature on the immune microenvironment in HCC was comprehensively analyzed.

**Results:** We found that AURKA was highly expressed in HCC, and it exhibited prognostic value. We selected eight AURKA-related genes with prognostic value through the protein-protein interaction network and successfully constructed a gene signature. The nine-gene signature could effectively stratify the risk of patients with HCC and demonstrated a good ability in predicting survival. The nomogram showed good discrimination and consistency of risk scores. In addition, the high-risk group showed a higher percentage of immune cell infiltration (i.e., macrophages, myeloid dendritic cells, neutrophils, and CD4+T cells). Moreover, the immune checkpoints SIGLEC15, TIGIT, CD274, HAVCR2, and PDCD1LG2 were also higher in the high-risk group versus the low-risk group.

**Conclusions:** This gene signature may be useful prognostic markers and therapeutic targets in patients with HCC.

## Introduction

According to global data, hepatocellular carcinoma (HCC) is the most common primary liver tumor and the third most common risk factor for cancer-related deaths worldwide (Sung et al., 2021). In 2020, there were approximately 906,000 newly diagnosed patients with liver cancer worldwide and approximately 830,000 liver cancer-related deaths (Sung et al., 2021). The main causes of liver cancer include hepatitis virus infection, smoking, alcoholic cirrhosis, chemical drugs, and aflatoxin infection (Forner et al., 2018). Approximately 400,000 people die annually in China, accounting for >50% of liver cancer-related deaths globally (Chen et al., 2016). Early-stage HCC is insidious and difficult to detect; consequently, a large number of patients already have advanced-stage disease at the time of diagnosis. At present, liver cancer is mainly treated by surgical resection, supplemented by other methods, such as ablation therapy, targeted therapy, and immunotherapy (Hartke et al., 2017). However, even with timely intervention, the recurrence and mortality rates remain high due to the high degree of malignancy in liver cancer, rapid disease progression, and poor prognosis (Li et al., 2015) There are numerous markers used for predicting the prognosis of patients with liver cancer; nevertheless, their effectiveness is currently limited. Therefore, there is an urgent need to identify more effective biomarkers for predicting the prognosis of patients with liver cancer.

Aurora kinase A (AURKA) is a member of the Aurora kinase family, which consists of AURKA A, B, and C (Carmena and Earnshaw, 2003). Human AURKA is located on chromosome 20q13 and encodes a protein of 403 amino acids. It mainly regulates mitotic spindle formation, stability, and chromosome segregation, and plays an important role in cell cycle regulation (Lindon et al., 2016). The abnormal expression of AURKA can lead to chromosomal abnormalities and instability of the cell genome, which is a risk factor for tumor formation (Wu et al., 2018). AURKA is abnormally expressed in a variety of tumors and regulates tumor proliferation, migration, invasion, and metastasis (Yan et al., 2016) In addition, it is involved in multiple signaling pathways, such as the TP53 pathway, Ras/mitogen-activated protein kinase (MAPK) pathway and NFKB pathway (Katayama et al., 2004; Briassouli et al., 2007; Umstead et al., 2017). Previous studies have confirmed that AURKA is related to the prognosis of a variety of cancers (breast, colorectal, pancreatic, gastric, and head and neck) and may be a therapeutic target (Jeng et al., 2004; Reiter et al., 2006; Zhang et al., 2015).

Investigations showed that AURKA played an important role in liver cancer progression. Jeng et al. confirmed that AURKA was overexpressed frequently and correlated with high grade and high stage in HCC (Jeng et al., 2004). Lu et al. reported that AURKA mediated c-Myc’s oncogenic effects in HCC (Lu et al., 2015). Zhang et al. revealed AURKA promoted chemoresistance through targeting NF-kappaB/microRNA-21/PTEN signaling pathway in HCC (Zhang et al., 2014). Chen et al. suggested that AURKA promoteed cancer metastasis through regulating epithelial-mesenchymal transition and cancer stem cell properties in HCC (Chen et al., 2017). However, the specific mechanism of AURKA in HCC is still not very clear and needs to be further explored.

Thus far, no study investigated the role of AURKA gene and AURKA-related prognostic genes in liver cancer. It is well established that the tumor immune microenvironment influences tumor progression (Locy et al., 2018). Currently, the immunological value of AURKA in liver cancer has not been reported. In this study, we first analyzed its clinical value in HCC and selected prognostic genes associated with AURKA. Furthermore, we developed an AURKA-related gene signature in HCC. Next, we constructed a nomogram by combining risk scores and clinical characteristics. Finally, we evaluated the relationship between the gene signature and tumor immunity in HCC.

## Methods

### Identification of AURKA as a Differentially Expressed Gene (DEG)

The RNA-seq data of LIHC patients from The Cancer Genome Atlas (TCGA, http://gdc.cancer.gov/) database and three datasets, including GSE14323 (HCC, *n* = 55; normal, *n* = 60), GSE14520 (HCC, *n* = 225; normal, *n* = 220) and GSE25097 (HCC, *n* = 268; normal, *n* = 289), from Gene Expression Omnibus (GEO, https://www.ncbi.nlm.nih.gov/geo/) database was downloaded to analysis the AURKA expression in LIHC patients. Gene expression levels were normalized by Robust Multi-Array Average (RMA).

**FIGURE 1:**
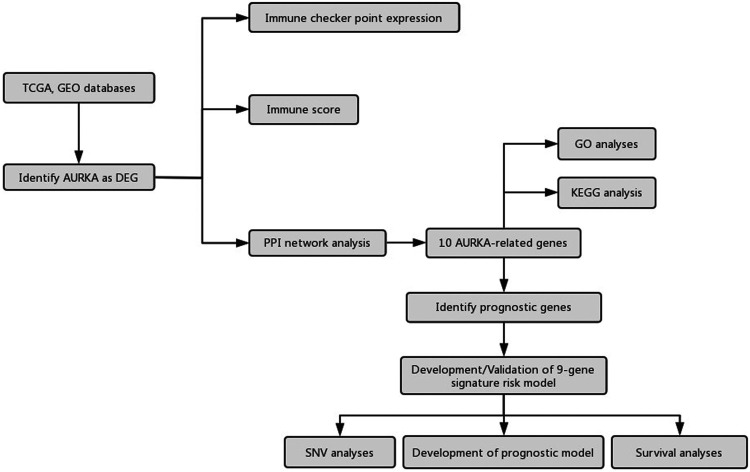
Flow chart of the study.

Differential gene expression analysis of mRNAs was performed based on TCGA database. Limma package of R software (R version 3.6.2) was used to conduct differential gene expression analysis. The adjusted *p*-value was analyzed to correct for false positive results in TCGA or GTEx. “Adjusted *p* < 0.05 and Log (Fold Change) >1 or Log (Fold Change) <−1” were regarded as the thresholds of differential expression of mRNAs. The study flowchart was presented in Figure 1.

### Protein-Protein Interaction Network and Gene Enrichment Analysis

STRING (version 11.0; http://string.embl.de/) is an open-access biological database that predicts comprehensive interactions of genes at the protein level from multiple organisms including Homo sapiens (Mering et al., 2003). To screen AURKA-related genes, we used STRING to explore AURKA-related genes as well as conduct PPI network analysis on AURKA-related genes. The protein–protein interactions (PPI) with medium confidence >0.4 were regarded as significant.

To further confirm the underlying function of potential targets, the data were analyzed by functional enrichment. Gene Ontology (GO) is a widely-used tool for annotating genes with functions, especially molecular function (MF), biological pathways (BP), and cellular components (CC). Kyoto Encyclopedia of Genes and Genomes (KEGG) Enrichment Analysis is a practical resource for analytical study of gene functions and associated high-level genome functional information. ClusterProfiler package (version: 3.18.0) in R was employed to analyze the GO function of potential targets and enrich the KEGG pathway. *p* < 0.05 was set as the cut-off criterion.

### Identification of Prognostic Genes

Gene Expression Profiling Interactive Analysis (GEPIA) is an interactive web application based on The Cancer Genome Atlas (TCGA) and Genotype-Tissue Expression databases (Tang et al., 2017). We used the Gene Expression Profiling Interactive Analysis to screen the prognostic value of AURKA and AURKA-related genes. The identification of prognostic genes was based on the following criteria: *1*) significant differences in gene expression levels between normal liver samples and liver tumor samples and *2*) significant association of the gene with both overall survival (OS) and disease-free survival (DFS) of patients with liver hepatocellular carcinoma (LIHC). *p*-values <0.05 denoted statistically significant differences.

### Development and Validation of the Gene Signature

Primary screening of the LIHC data from TCGA database was performed for missing data. After deleting the samples with missing data, The available LIHC data from the TCGA database (340 samples) was divided into two subsets: a training set (*n* = 240) and a validation set (*n* = 100) randomly using the random function of Microsoft Excel program. The training set was used to train a predictive model and the test set was applied for validation. Another database International Cancer Genome Consortium (ICGC) (dcc.icgc.org) was also used as validation set.

Identified prognostic genes were submitted to the multivariate Cox regression model to calculate each prognostic gene’s coefficient and risk scores. We used X-tile plot (version 3.6.1, http://www.tissuearray.org/rimmlab) to determine the optimum cutoff of AURKA-related gene signature risk score. X-tile which could calculate the best cut-point of sub-populations, is a software developing by team Rimm Laboratory from Yale University (Camp et al., 2004). A train set and two validation sets were divided into three sub-groups using the same best cut-off value. Risk score analysis, including risk score distribution, survival status, and gene expression heatmap among sub-groups were performed. Kaplan–Meier curves were analyzed using Kruskal-Wallis test and visualized through GraphPad Prism (version 8.0). Receiver operating characteristic (ROC) curve analysis was performed to assess the predictive value of AURKA-related gene signature. The clinical characteristics of three cohorts were shown in Table 1. The correlation among clinicopathological characteristics and risk groups were analyzed by the chi-square test. For sample sizes of less than 40 or theoretical frequencies (T) of less than 1, Fisher exact probability method was used.

**TABLE 1:** Clinical characteristics of HCC cohorts.

Clinical features		Training set (*n* = 240)	Validation set (*n* = 100)	ICGC-LIRI-JP (*n* = 243)
Age	<50	45	21	15
	≥50	195	79	228
Gender	Female	80	28	61
	Male	160	72	182
T	Early (T1+T2)	176	76	-
	Late(T3+T4)	63	23	-
	Unknown	1	1	-
N	N0	172	67	-
	N1+N2	2	1	-
	Unknown	66	32	-
M	M0	171	73	-
	M1	1	2	-
	Unknown	68	25	-
Stage	Stage I/II	166	71	146
	Stage III/IV	60	23	97
	Unknown	14	6	0
Grade	G1	38	14	-
	G2	114	47	-
	G3	76	35	-
	G4	9	3	-
	Unknown	3	1	-
Recurrence	yes	134	44	-
	no	106	56	-
RiskGroup	low risk	135	49	199
	mid risk	78	40	37
	high risk	27	11	7
Status	Alive	148	69	199
	Dead	92	31	44

### Distribution of Somatic Mutations

To identify the somatic mutations of patients with LIHC, we downloaded single-nucleotide polymorphism (SNV) data and clinical follow-up information from TCGA database. The downloaded single-nucleotide polymorphism data were organized in the multiple alignment (MAF) format and visualized using the “maftools” package in R software. The horizontal histogram showed the genes with the highest frequency of mutation.

### Immune Infiltration Analysis

To explore the associations between different subgroups and immune cells infiltration, we employed Tumor Immune Estimation Resource (TIMER), which is a useful resource for comprehensive analysis of tumor-infiltrating immune cells (Li et al., 2017). The infiltration of six type of immune cell, including B cell, Macrophage, Myeloid dendritic cell, Neutrophil, T cell CD4^+^ and T cell CD8^+^, were calculated. SIGLEC15, IDO1, CD274, HAVCR2, PDCD1, CTLA4, LAG3 and PDCD1LG2 were selected to be immune checkpoints and the expression values of these eight immune checkpoints among sub-groups were explored. Differences between the three groups were assessed using the Kruskal-Wallis test. *p* < 0.05 was considered statistically significant. All the above analysis methods and R package were implemented by R foundation for statistical computing (2020) version 4.0.3 and software packages ggplot2 and pheatmap.

### Construction and Validation of a Prognostic Model

A nomogram model was constructed to predict the probability of survival at 3 and 5 years for liver cancer patients. Briefly, the prognostic value of the clinicopathological characteristics for OS was estimated through univariate and multivariate Cox regression analyses in both training set and validation set. The performance of the risk model was validated by internal validation and external validation. Internal validation was performed by bootstrap Cox proportional regression analysis based on 1,000 bootstrap samples. Validation set was conducted based on another HCC patients from the TCGA database. Those parameters with *p*-values <0.05 in both training set and validation set were identified as potential prognostic factors, which were were included in multivariate Cox regression model and visualized using R package “rms.” The Calibration curves were plotted to analyze the diagnostic performance of the nomogram. The ROC curve were conducted to determine the clinical value of the nomogram.

## Results

### AURKA mRNA Level in HCC Samples

**FIGURE 2:**
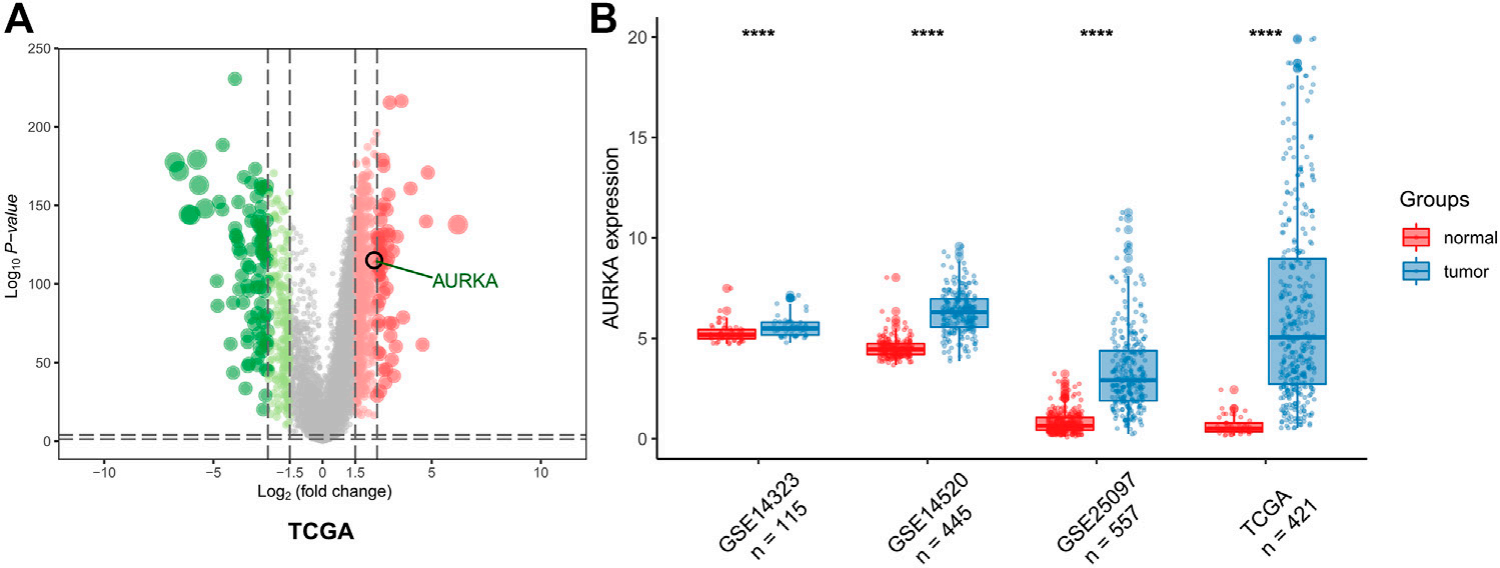
AURKA expression. **(A)** Volcano plot of all DEGs. AURKA gene was identified. **(B)** Histogram of GSE14323, GSE14520 and GSE25097.

The results of the DEG analysis are shown in Figure 2. In TCGA database, a total of 421 HCC samples were selected for the DEG analysis. The analysis showed that the AURKA gene was upregulated (log_2_ fold change >1.5 and adjusted *p*-value <0.05), which indicated that the mRNA expression levels of AURKA differ significantly between normal liver tissue and liver cancer tissue. The mRNA expression levels of AURKA were also determined using three Gene Expression Omnibus data sets (GSE14323, *n* = 115; GSE14520, *n* = 445; and GSE25097, *n* = 557). The results also showed that AURKA was significantly highly expressed in HCC.

### PPI Network Analysis

**FIGURE 3:**
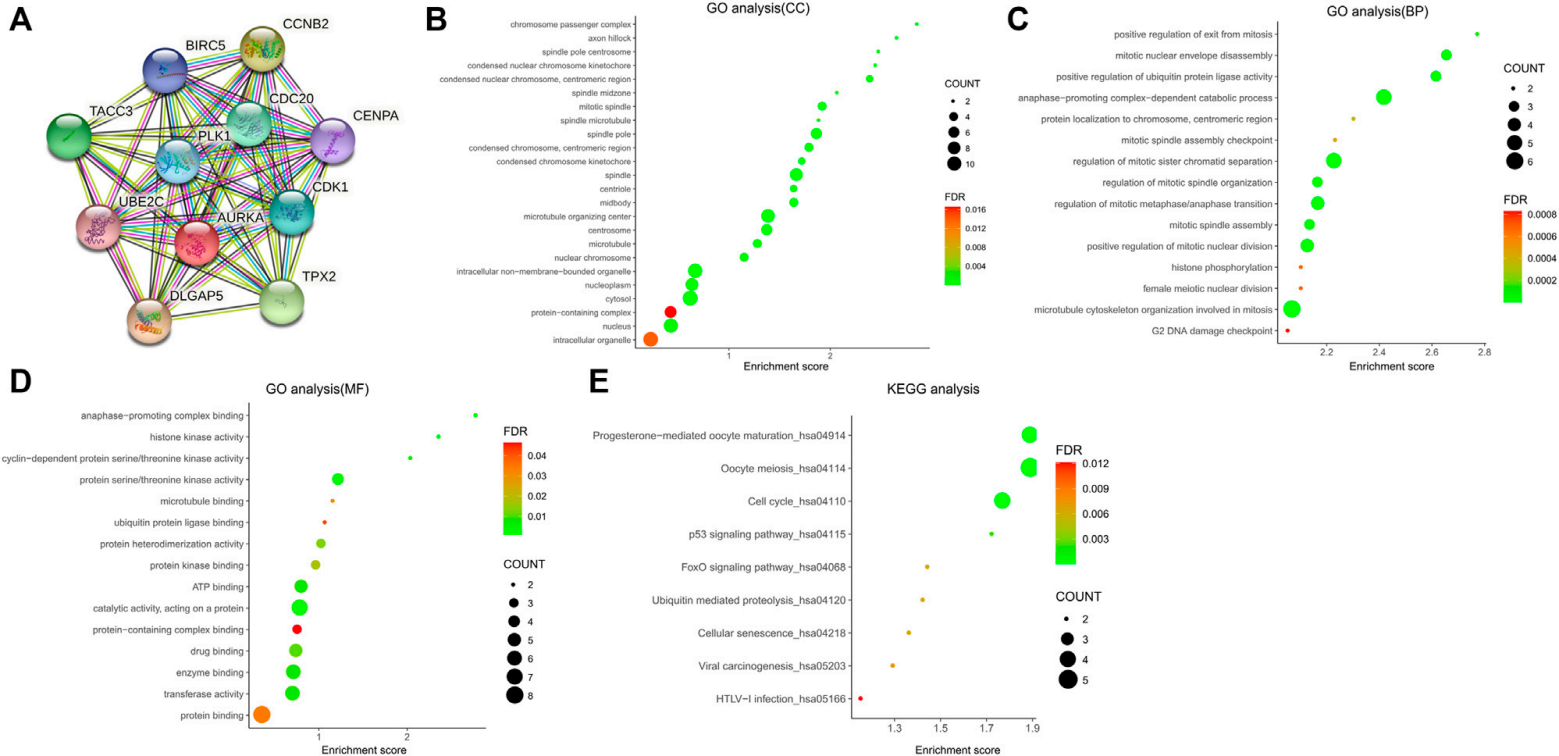
PPI network of AURKA and AURKA-related genes. **(A**–**D)** GO analysis of AURKA and AURKA-related genes. **(E)** KEGG pathway analysis of AURKA and AURKA-related genes.

According to the predictive results of the Search Tool for the Retrieval of Interacting Genes (STRING) database, another ten genes were identified as AURKA-related genes with significant interaction, namely targeting protein for Xklp2 (TPX2), cyclin dependent kinase 1 (CDK1), polo like kinase 1 (PLK1), DLG associated protein 5 (DLGAP5), cell division cycle 20 (CDC20), baculoviral IAP repeat containing 5 (BIRC5), transforming acidic coiled-coil containing protein 3 (TACC3), centromere protein A (CENPA), cyclin B2 (CCNB2), and ubiquitin conjugating enzyme E2 C (UBE2C). The protein-protein interaction (PPI) network of AURKA and AURKA-related genes was constructed and visualized using the online STRING database (Figure 3A).

The Gene Ontology (GO) enrichment analysis was composed of three parts: GO biological process (GO-BP), GO cellular component (GO-CC), and GO molecular function (GO-MF). In GO-CC (Figure 3B), these genes were enriched in condensed nuclear chromosome, centromeric region, mitotic spindle, and spindle pole. In GO-BP (Figure 3C), AURKA and its related genes were significantly enriched in mitotic nuclear envelope disassembly, positive regulation of ubiquitin protein ligase activity, and anaphase-promoting complex-dependent catabolic process. In GO-MF (Figure 3D), genes were mainly enriched in protein serine/threonine kinase activity, protein heterodimerization activity, and protein kinase binding. For the Kyoto Encyclopedia of Genes and Genomes pathway analysis, nine pathways (Figure 3E) were observed, namely progesterone-mediated oocyte maturation, oocyte meiosis, cell cycle, TP53 signaling pathway, forkhead box O (FOXO) signaling pathway, ubiquitin-mediated proteolysis, cellular senescence, viral carcinogenesis, and human T-lymphotropic virus type I (HTLV−I) infection.

### Development and Validation of a Nine-Gene Signature

**FIGURE 4:**
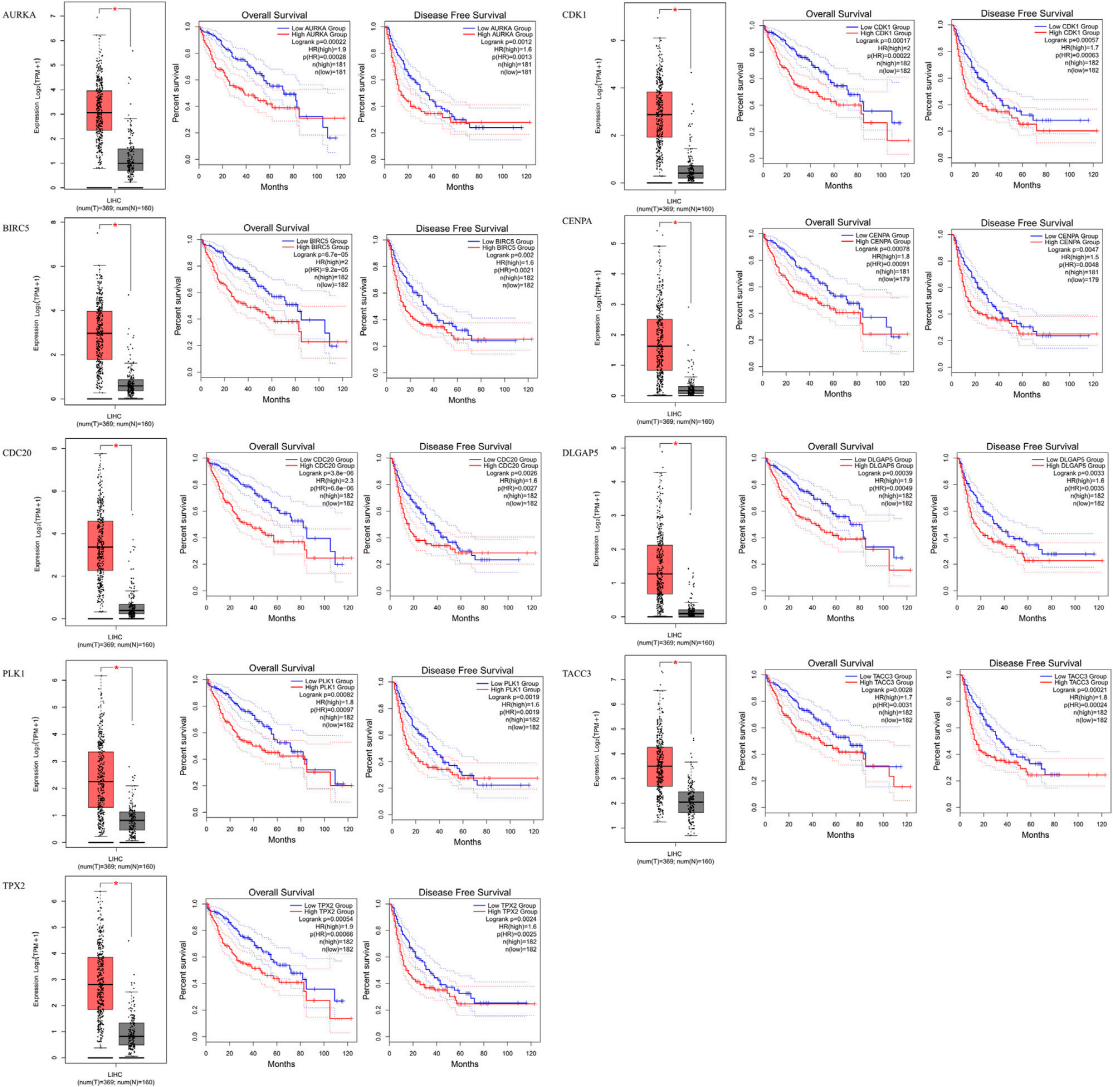
Identification of prognostic genes. AURKA, BIRC5, CDC20, PLK1, TPX2, CDK1, CENPA, DLGAP5 and TACC3 gene expression levels were significantly higher in HCC patients, and these nine genes are related to overall survival and disease-free survival in HCC.

The results of the identification of prognostic genes are shown in Figure 4. According to the screening strategy and criteria described above, two AURKA-related genes, namely CCNB2 (OS Log-rank *p* > 0.05) and UBE2C (OS Log-rank *p* > 0.05), were excluded. Eight other AURKA-related genes were significantly highly expressed in HCC tissue compared with normal liver tissue and correlated with prognosis.

The AURKA and the other eight AURKA-related genes were used to construct a risk model. The risk score was calculated through multivariate regression analyses. The cutoff value of the risk score was identified using the X-tile software. According to the results, the HCC samples were divided into three subgroups. The cutoff values of the risk score were 0.17 and 0.95. HCC samples with a risk score <0.17 and >0.95 classified into the low- and high-risk groups, respectively. The remaining samples were assigned to the moderate-risk group.

**FIGURE 5:**
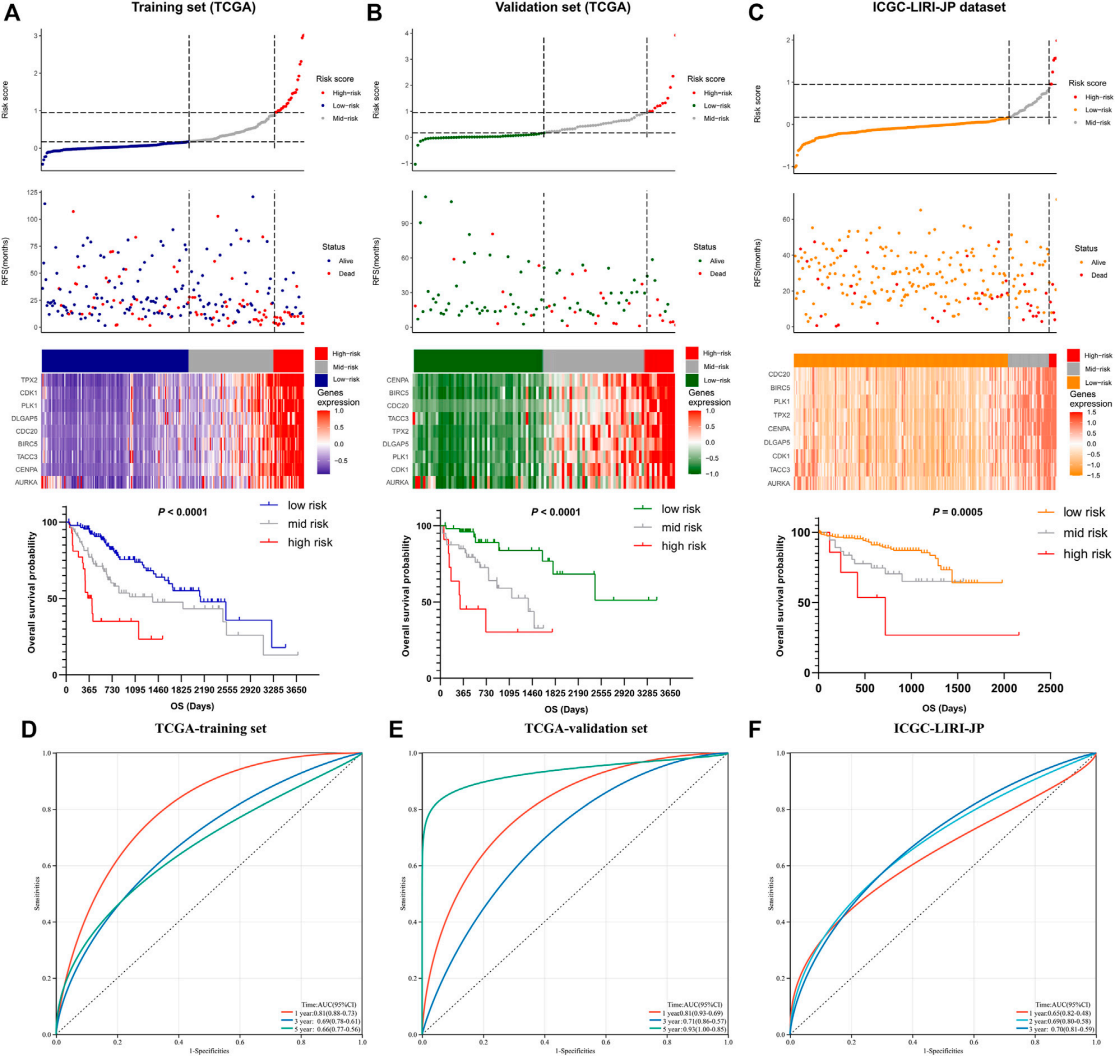
The prognostic significance of the nine-gene risk scoring model. **(A)** The distribution of risk scores and survival status of liver cancer patients in the training set. HCC Patients with a higher risk score have a lower overall survival rate. **(B**,**C)** The distribution of risk scores and survival status of liver cancer patients in validation set. A higher risk score in HCC patients also have a lower overall survival rate. **(D**–**F)** The ROC curves of the training set and two validation sets.

The details of the risk model showed in Figure 5, which revealed that higher risk scores were associated with higher expression levels of the nine-gene signature. Furthermore, higher risk scores also indicated worse OS. These results were similar in both the training and other two validation sets. Collectively, these results suggest that the risk model had potential value in predicting the prognosis of HCC. Furthermore, as the ROC curves showed, the area under the ROC curve (AUC) of the training set and two validation sets were higher than 0.5, which indicated that AURKA-related gene signature risk model had important value in predicting prognosis (Figures 5D–F).

The clinicopathological characteristics among the risk groups in training set were shown in Table 2. Age, T stage, pathological TNM (pTNM) stage, grade, recurrence, and survival endpoint were significantly different between the three risk groups (*p* < 0.05).

**TABLE 2:** Correlation among risk groups with clinical features in training set.

Clinical features		Risk group			*p* value
		Low	Mid	High	
Age	<50	23	12	10	0.0339
	≥50	112	66	17	
Gender	Female	42	29	9	0.6639
	Male	93	49	18	
T	Early (T1+T2)	113	52	11	<0.001
	Late (T3+T4)	21	26	16	
N	N0	90	59	23	1
	N1+N2	1	1	0	
M	M0	90	58	23	1
	M1	1	0	0	
Stage	Stage I/II	106	49	11	<0.001
	Stage III/IV	21	24	15	
Grade	G1+G2	98	45	9	0.0002
	G3+G4	36	31	18	
Recurrence	Yes	72	23	11	0.0031
	No	63	55	16	
Status	Alive	96	42	10	0.0009
	Dead	39	36	17	

### Somatic Mutation Results

**FIGURE 6:**
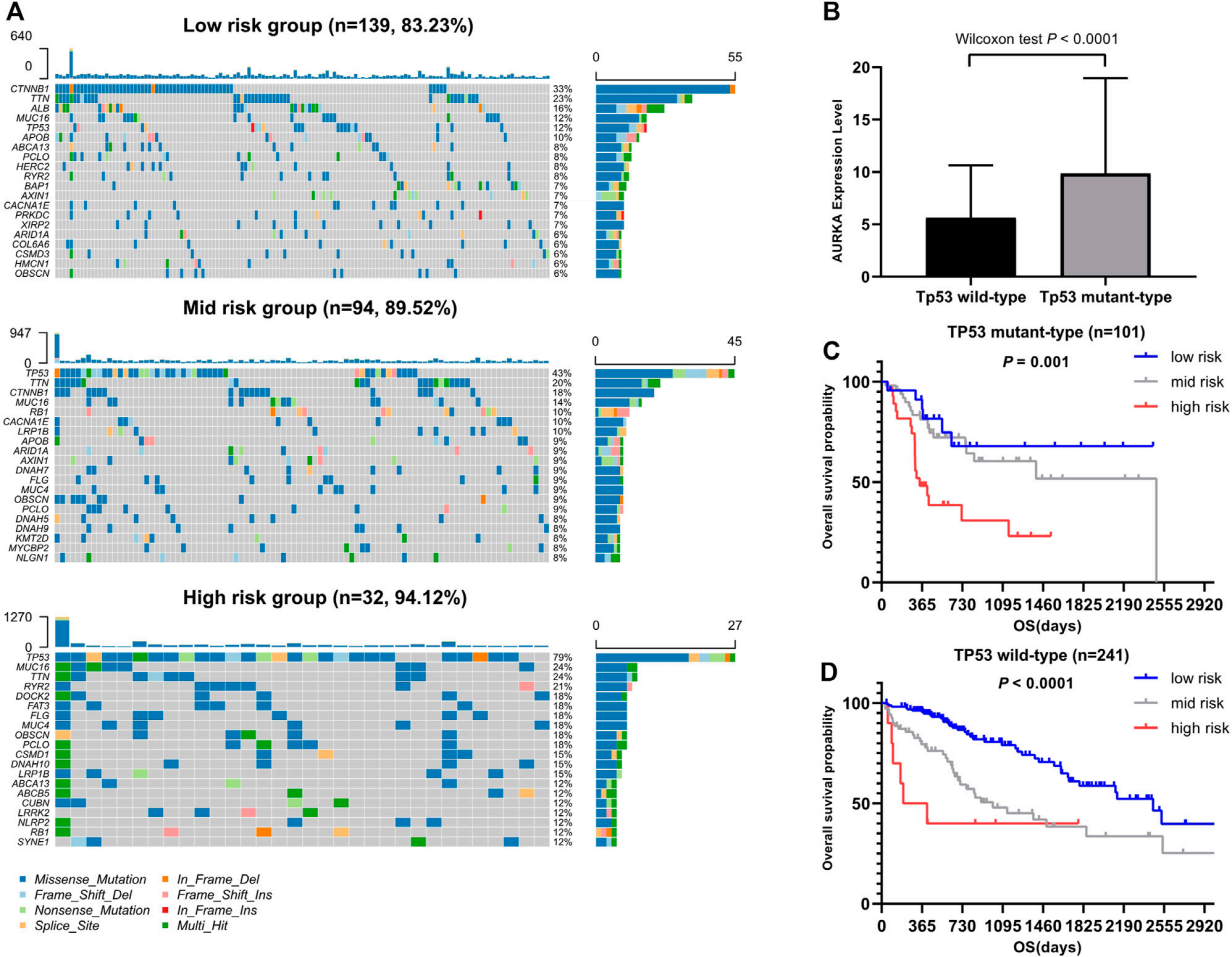
Association between somatic mutation and risk score. **(A)** Overview of somatic mutations in different risk groups. **(B)** Comparison of AURKA expression between TP53 mutation group and Tp53 wild group. **(C)** Kaplan-Meier survival analysis of the different risk groups of TP53 mutation. **(D)** Kaplan-Meier survival analysis of the different risk groups of Tp53 wild.

Figure 6 illustrates the somatic landscape of the three risk subgroups. Information on the mutation status of each gene in each sample was shown in the waterfall plot, where different colors with specific annotations at the bottom indicated the various types of mutation. The barplot above the legend exhibited the number of mutations. The results showed that CTNNB1 was the most commonly mutated gene in the low-risk group. Tumor protein p53 (TP53) was the most frequently mutated gene in both the moderate- and high-risk groups. Hence, we further grouped the HCC samples into two groups based on the TP53 mutation status. A total of 101 and 241 HCC samples were assigned to the TP53 mutant- and wild-type groups, respectively. The survival analyses of both the TP53 mutant- and wild type cohorts yielded similar results. Higher risk scores were associated with worse prognostic outcome (*p* < 0.05).

### Association Between Risk Score and Immune Infiltration

**FIGURE 7:**
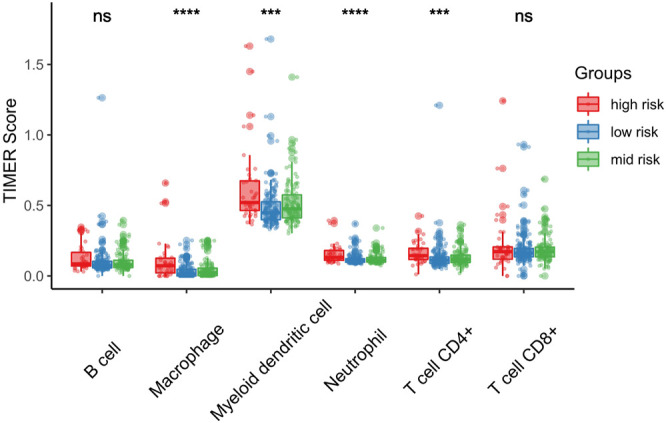
Association between risk score and immune infiltration. Macrophage, Myeloid dendritic cell, Neutrophil and CD4+T cell are significantly high in high risk score groups. *p < 0.05; **p < 0.01; ***p < 0.001.

**FIGURE 8:**
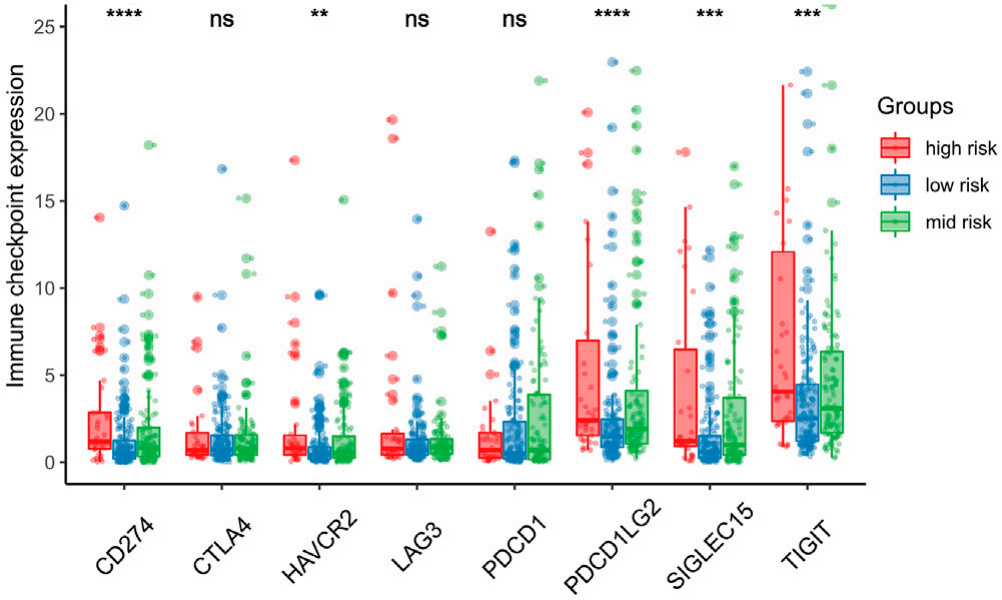
Association between risk score and immune checkpoint. SIGLEC15, TIGIT, CD274, HAVCR2 and PDCD1LG2 expression mainly expressed in the high-risk score group. *p < 0.05; **p < 0.01; ***p < 0.001.

We further investigated differences in the degree of immune infiltration in various risk groups. Using the TIMER database, we evaluated the immune cell infiltration in samples from the three aforementioned groups. As shown in Figure 7, we found that the level of immune cell infiltration (macrophages, myeloid dendritic cells, neutrophils, and CD4T cells) was significantly different between the three groups. To further investigate the levels of immune cell infiltration on the gene level, the following eight immune-related genes were selected: sialic acid binding Ig like lectin 15 (SIGLEC15), T cell immunoreceptor with Ig and ITIM domains (TIGIT), CD274, hepatitis A virus cellular receptor 2 (HAVCR2), programmed cell death 1 (PDCD1), cytotoxic T-lymphocyte associated protein 4 (CTLA4), lymphocyte activating 3 (LAG3), and programmed cell death 1 ligand 2 (PDCD1LG2). We compared the expression levels of immune-related genes among the three risk groups. The results are shown in Figure 8. Except for CTLA4, LAG3, and PDCD1, the expression levels of the other five immune-related genes differed significantly between the three groups. Overall, the high-risk group showed significantly higher levels of immune gene expression and immune cell infiltration compared with the other groups.

### Construction and Evaluation of the Nomogram Model

As shown in Table 3, we performed the univariate and multivariate analyses using the SPSS software (version 23.0; IBM Corporation, Armonk, NY, United States) to identify independent prognostic factors predicting OS in patients with HCC. In the training data set, the risk score, T stage, M stage, pTNM stage, and recurrence were identified as independent prognostic factors. The results of the testing and training data sets were similar. Overall, the risk score, T stage, and pTNM stage were identified as independent prognostic factors in both data sets.

**TABLE 3:** Univariate and multivariate Cox regression analyses of risk factors associated with overall survival.

HCC cohorts		Univariable analyses				Multivariable analyses		
		*p*	HR	95.0% CI		*p*	HR	95.0% CI
Validation set	Age	0.418	1.012	0.984–1.041				
	Gender	0.149	0.592	0.29–1.207			
	T stage	0	4.017	1.925–8.38		0	8.257	2.168–31.451
	N stage	0.04	8.998	1.107–73.152		0.908	1.143	0.118–11.109
	M stage	0.088	3.602	0.825–15.724		0.259	4.284	0.343–53.561
	pTNM_stage	0	4.602	2.114–10.017		0.003	5.318	1.778–15.907
	grade	0.205	0.601	0.273–1.322			
	recurrence	0.635	1.193	0.575–2.476			
	Low risk group	0.001				0.019		
	Mid risk group	0.005	3.936	1.515–10.224		0.033	5.677	1.151–27.994
	High risk group	0	8.064	2.687–24.201		0.005	11.994	2.119–67.908
Training set								
	Age	0.399	1.007	0.991–1.023			
	Gender	0.65	0.906	0.592–1.387			
	T stage	0	2.428	1.595–3.697		0.028	1.968	1.078–3.594
	N stage	0.824	1.253	0.173–9.092		0.517	1.95	0.258–14.738
	M stage	0.012	13.563	1.763–104.315		0.003	26.515	2.979–236.021
	pTNM_stage	0	2.281	1.464–3.554		0	2.281	1.464–3.554
	grade	0.17	1.349	0.88–2.068			
	recurrence	0.016	1.754	1.111–2.769		0.048	1.967	1.005–3.851
	Low risk group	0				0		
	Mid risk group	0.009	1.842	1.169–2.905		0.084	1.854	0.92–3.734
	High risk group	0	4.85	2.702–8.706		0	5.262	2.311–11.981

**FIGURE 9:**
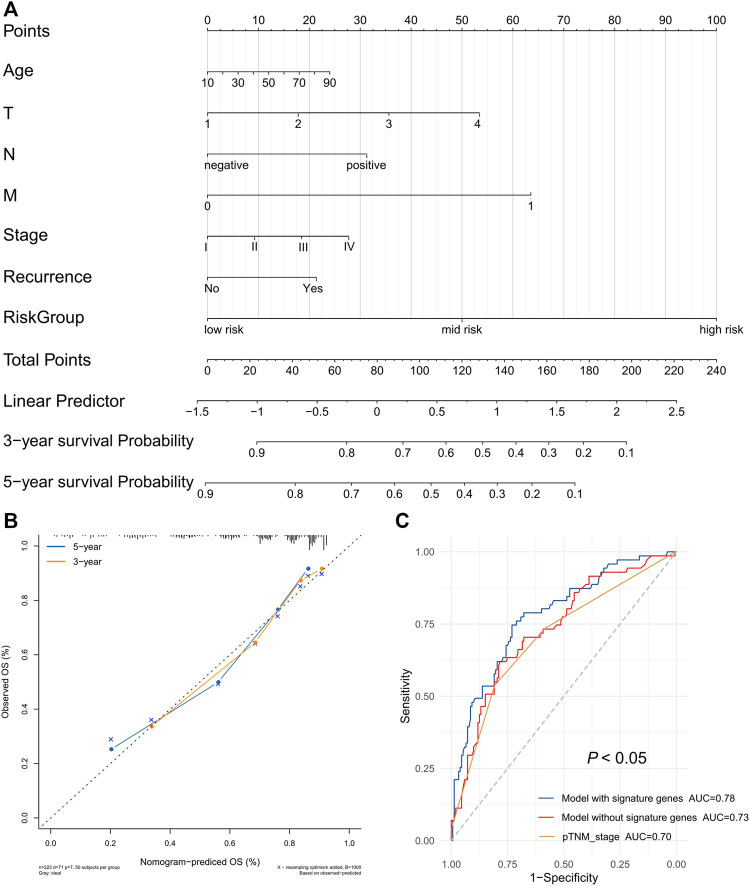
The prognostic nomogram with the risk score in HCC. **(A)** A nomogram for predicting 3- and 5-year survival possibilities of HCC. **(B)** The calibration curve of 3-year and 5-year survival. **(C)** Time-dependent receiver operating characteristic (ROC) curves for gene signature and TNM stage.

Hence, we used age, T stage, N stage, M stage, pTNM stage, recurrence, and risk group as estimated factors in the construction of our model. A nomogram was constructed to estimate the probabilities for three- and 5-year survival. Calibration curves analysis showed that the new nomogram model had good predictive accuracy (Figure 9B). Furthermore, the new nomogram model achieved an area under the curve (AUC) of 0.78 at 5 years, which was better than that of a model without the gene signature (AUC = 0.73) or pTNM stage (AUC = 0.70) (Figure 9C).

## Discussion

The prognosis of patients with HCC varies greatly. The 5-year survival rate after resection of early HCC can be as high as 70%, whereas that of patients with vascular invasion or advanced HCC is markedly lower (Mazzaferro et al., 2011; Grandhi et al., 2016). Therefore, the use of effective prognostic indicators can promptly identify high-risk patients and assist in implementing individualized treatment to improve the prognosis.

In the present study, we found that the AURKA gene was highly expressed in HCC and an independent prognostic risk factor. The GO analysis of AURKA and related genes indicated that these genes are involved in a variety of biological process, including nuclear chromosome condensation, centromeric regions, mitotic spindle and spindle poles, mitotic nuclear envelope disassembly, and ubiquitin protein ligase activity. Kyoto Encyclopedia of Genes and Genomes (KEGG) pathway analysis showed that AURKA and related genes regulate the progression of HCC through multiple pathways, such as the TP53 signaling pathway and FOXO signaling pathway. Numerous studies have demonstrated that the TP53 signaling pathway is involved in the development of a variety of tumors and plays a regulatory role in tumor immunity (Stegh, 2012; Blagih et al., 2020; Muñoz-Fontela et al., 2016). The role of the FOXO signaling pathway in tumors has also received extensive attention (Farhan et al., 2017; Coomans de Brachène and Demoulin, 2016). In general, the enrichment analysis revealed some potential mechanisms and possible pathways of AURKA and its related genes in HCC. In addition, it provided some new ideas for the treatment of patients with HCC.

Among the nine genes associated with AURKA, eight genes (BIRC5, CDC20, PLK1, TPX2, CDK1, CENPA, DLGAP5, and TACC3) were identified as prognostic genes in HCC. Therefore, we used these nine genes to construct the AURKA-related gene signature for the prediction of prognosis of patients with liver cancer. In gastric cancer, high expression of BIRC5 promotes gastric cancer metastasis and is associated with poor prognosis (Zou et al., 2019). However, in lung cancer, high expression of BIRC5 may prolong OS and DFS (Vischioni et al., 2004). In pancreatic cancer, CDC20 can promote tumor cell proliferation and affect the progression of pancreatic cancer (Chang et al., 2012). CDK1 is considered a synthetic target for KRAS-mutated tumors and has been identified as a prognostic marker in numerous types of cancer (Sung et al., 2014; Costa-Cabral et al., 2016). CENPA plays a key role in cell mitosis. Of note, it is abnormally expressed in tumors and regulates tumor cell activity (Valdivia et al., 2009). DLGAP5 expression is regulated by the ubiquitin-proteasome pathway and participates in tumor cell migration and invasion (Hsu et al., 2004). PLK1 is a key regulator of mitosis and is involved in multiple stages of mitosis (De et al., 2018). Downregulation of PLK1 can inhibit the invasion and metastasis of esophageal cancer cells (Li et al., 2014). In colorectal cancer, PLK1 may promote the growth, invasion, and metastasis of colorectal cancer cells through the PDKI-PLK1-MYC signaling pathway (Tan et al., 2013). TACC3 can activate the Akt/RAS/mitogen-activated protein kinase kinase/extracellular signal–regulated kinase (Akt/RAS/MEK/ERK) signaling pathway to promote the malignant transformation of cells (Burgess et al., 2018). High TACC3 expression has been found in a variety of tumors and is closely related to poor prognosis (Wang et al., 2017). TPX2 is mainly involved in centrosomal maturation and spindle formation (Gruss and Vernos, 2004). In gliomas, MiR-1294 can target TPX2 to inhibit tumor cell proliferation and enhance sensitivity to chemotherapy (Chen et al., 2018). Several previous studies have successfully constructed multi-gene signatures for risk stratification and prognosis prediction in HCC (Zhou et al., 2020; Ouyang et al., 2020). In this study, we constructed a new nine-gene signature to predict the prognosis of patients with liver cancer. This gene signature was verified using an internal verification data set. Gene signatures can effectively classify patients into high-, moderate-, and low-risk groups. Higher risk scores in the training and validation data sets indicated a poor prognosis in HCC. Finally, we constructed a personalized nomogram based on the risk scores, with a concordance index of 0.78.

As an important tumor suppressor gene, TP53 plays a vital role in cell cycle regulation. TP53 mutation is a common mutation in tumors and the most important mutation in liver cancer. This mutation can promote the proliferation, migration, and invasion of tumor cells and increase resistance to drugs (Warren et al., 2013). We divided the HCC cohort of TCGA data set into two groups (TP53 mutation and wild type) and investigated the relationship between the gene signature and these two groups. We found that the gene signature could effectively predict the risk of patients in the TP53 mutation group, thereby effectively classifying these patients into low-, moderate-, and high-risk groups. Numerous recent studies have also confirmed the close relationship between TP53 mutation and tumor immunity (Long et al., 2019; Wu et al., 2020; Sun et al., 2020). Based on the immunoprognostic model established by TP53 mutation, Long et al. found that the levels of T cell follicular helper proteins, T cell regulatory proteins, and macrophages M0 were higher in the high-risk HCC group versus the other groups (Long et al., 2019). Notably, the expression of immune checkpoints CTLA4, PDCD1, and T-cell immunoglobulin mucin family member 3 (TIM3) were also higher in the high-risk group. The investigators suggested that TP53 mutations significantly reduced the immune response in liver cancer.

Immune cell infiltration affects tumor progression. Numerous immunotherapies have been used to regulate immune cells in tumors. Therefore, we investigated the immune cell infiltration in different risk groups. We found that the numbers of macrophages, myeloid dendritic cells, neutrophils, and CD4^+^ T cells differed significantly in different risk score groups. Higher risk scores were linked to higher numbers of these four types of immune cells. Macrophages play a dual role in the tumor microenvironment, promoting tumor formation and development as well as inhibiting tumor growth (Kim and Bae, 2016). It has been confirmed that the degree of macrophage infiltration in the tumor microenvironment is related to prognosis (Conway et al., 2016). It is currently thought that neutrophils in the tumor microenvironment directly kill or stimulate the immune system to inhibit tumor cells and can also promote immune escape of tumor cells (Kim and Bae, 2016). Myeloid dendritic cells mainly play an antigen-presenting role to activate T cells and induce immune responses (Garris and Luke, 2020). CD4^+^ T cells mainly support CD8^+^ T toxic lymphocytes and enhance their anti-tumor immune effect (Ghiringhelli et al., 2006). Our results showed differences in the distribution of immune cells in the tumor microenvironment among the different risk groups and revealed the underlying reason for the poor prognosis observed in the high-risk group. To the best of our knowledge, this is the first study to investigate the relationship between AURKA and related genes and tumor immunity.

Immune checkpoint inhibitors are a new approach to the treatment of tumors. Several immune checkpoint inhibitors have been used effectively in the treatment of liver cancer (Liu and Qin, 2019). In this study, we also assessed the relationship between the risk score and immune checkpoints. The results found that the expression of the five immune checkpoints (SIGLEC15, TIGIT, CD274, HAVCR2, and PDCD1LG2) varied in different risk groups. Higher risk scores were associated with higher expression of the five immune checkpoints. These five immune checkpoints play an important role in the activation of T cells. PDCD1LG1 and PDCD1LG2 are the two ligands of PDCD1. In the tumor microenvironment, PDCD1 on the surface of immune cells binds to the PDCD1LG1 and PDCD1LG2 receptors on the surface of tumor cells to activate a series of signal factors in immune cells. This process initiates a series of signaling factors in immune cells to inhibit T cell activation and promote T cell failure, thus helping tumor cells to evade immunosurveillance (Barclay et al., 2018; Ai et al., 2020). SIGLEC15 is a newly discovered immune checkpoint. Wang et al. reported that high expression of SIGLEC15 in tumors can significantly inhibit the activity of T cells (Wang et al., 2019). Furthermore, the inhibition or knockout of SIGLEC15 expression can improve the anti-tumor ability of T cells in mice (Wang et al., 2019). HAVCR2 is thought to play a dual role, inducing immune tolerance and promoting tumor cell apoptosis (Das et al., 2017). TIGIT is mainly expressed on T cells and natural killer cells. Joller et al. observed significant T cell proliferation in TIGIT-knockout mice (Joller et al., 2011). The expression of these immune checkpoints significantly affects tumor prognosis. At present, the use of single immune checkpoint blockers or combinations of these agents has shown good efficacy in different tumors (Darvin et al., 2018).

This study is characterized by several limitations. Firstly, all analyses were based on public databases. The specific mechanisms of AURKA and related genes in liver cancer have not been thoroughly investigated. Furthermore, the gene signature was associated with immune infiltration and immune checkpoint expression in HCC and affected the prognosis of patients with this disease. Further studies are needed to examine the value of gene signatures in immune invasion and prognosis in HCC.

In summary, we found that nine AURKA-related genes with prognostic value can be used as prognostic markers for liver cancer. The gene signature based on AURKA successfully classified patients with liver cancer into high-, moderate- and low-risk groups. Hence, the gene signature can may be an effective marker for the prognosis of HCC. In addition, the risk score was related to immune cell infiltration and immune checkpoint expression in HCC.

## Data Availability

The original data presented in the study are included in the article/Supplementary Material, further inquiries can be directed to the corresponding author.
